# Design and Framework of Non-Intrusive Spatial System for Child Behavior Support in Domestic Environments

**DOI:** 10.3390/s25175257

**Published:** 2025-08-23

**Authors:** Da-Un Yoo, Jeannie Kang, Sung-Min Park

**Affiliations:** 1 Department of Architecture, Ewha Womans University, Seoul 03760, Republic of Korea; 2 College of Art & Design, Ewha Womans University, Seoul 03760, Republic of Korea; herenow.kang@ewha.ac.kr; 3 Division of Electronic & Semiconductor Engineering, Ewha Womans University, Seoul 03760, Republic of Korea

**Keywords:** child-behavior, LiDAR, sensor, design strategy, behavior-guided system

## Abstract

This paper proposes a structured design framework and system architecture for a non-intrusive spatial system aimed at supporting child behavior in everyday domestic environments. Rooted in ethical considerations, our approach defines four core behavior-guided design strategies: routine recovery, emotion-responsive adjustment, behavioral transition induction, and external linkage. Each strategy is meticulously translated into a detailed system logic that outlines input conditions, trigger thresholds, and feedback outputs, designed for implementability with ambient sensing technologies. Through a comparative conceptual analysis of three sensing configurations—low-resolution LiDARs, mmWave radars, and environmental sensors—we evaluate their suitability based on technical feasibility, spatial integration, operationalized privacy metrics, and ethical alignment. Supported by preliminary technical observations from lab-based sensor tests, low-resolution LiDAR emerges as the most balanced option for its ability to offer sufficient behavioral insight while enabling edge-based local processing, robustly protecting privacy, and maintaining compatibility with compact residential settings. Based on this, we present a working three-layered system architecture emphasizing edge processing and minimal-intrusion feedback mechanisms. While this paper primarily focuses on the framework and design aspects, we also outline a concrete pilot implementation plan tailored for small-scale home environments, detailing future empirical validation steps for system effectiveness and user acceptance. This structured design logic and pilot framework lays a crucial foundation for future applications in diverse residential and care contexts, facilitating longitudinal observation of behavioral patterns and iterative refinement through lived feedback. Ultimately, this work contributes to the broader discourse on how technology can ethically and developmentally support children’s autonomy and well-being, moving beyond surveillance to enable subtle, ambient, and socially responsible spatial interactions attuned to children’s everyday lives.

## 1. Introduction

Advanced ambient sensing technologies have been transforming spatial design, enabling the creation of dynamic environments that not only serve as passive shelters but also act as active platforms to guide children’s behavior, accommodate emotional states, and foster autonomy. In recent years, interdisciplinary efforts from architecture, human–computer interactions (HCI), and user experience (UX) design have proposed sensory-friendly environments, particularly for neurodiverse populations [1,2]. These environments aim to regulate stress and create calming spatial cues through elements like lighting, acoustics, and material textures. However, many of these existing approaches, while valuable, often remained conceptual or demonstrative, primarily focusing on static sensory mitigation rather than dynamic behavioral adaptation. For instance, Mostafa’s (2008) pioneering work laid out spatial principles for autism-friendly architecture, emphasizing predictability, acoustic zoning, and visual cues [1]. Crucially, these strategies were predominantly static, lacking real-time response mechanisms to user behavior. More technologically adaptive approaches include Deng and Rattadilok (2022), who developed a sensor and a machine learning-based sensory management system for children with Autism-Spectrum-Disorder (ASD) that responds to physiological signals [3]. Similarly, Voss et al. (2019) introduced Superpower Glass, a wearable device providing social-emotional feedback to children with ASD via facial emotion recognition [4]. While these studies demonstrated the feasibility of emotionally responsive systems, their reliance on wearable devices or high-contact interventions limited their suitability for truly non-intrusive, space-embedded applications in domestic environments. Furthermore, the broader field of Human Activity Recognition (HAR) in smart living services has highlighted the critical need for solutions that balance context awareness, data availability, personalization, and privacy [5].

The emergence of non-visual and privacy-preserving sensing technologies, such as low-resolution LiDAR and mmWave radar, offers a promising pathway to overcome these limitations. These technologies can capture rich behavioral data without infringing on privacy, making them ideal for sensitive environments like homes. Indeed, the potential of technology to monitor health and behavior in children, while driven by best intentions, requires careful consideration of ethical implications and potential unintended consequences [6,7]. However, despite advancements in sensor capabilities, a significant gap remains in translating these technical potentials into holistic, ethically aligned spatial systems that dynamically support child behavior in everyday domestic life.

This paper addresses a critical gap in this field, i.e., the absence of spatial systems that effectively translate behavioral cues into adaptive, real-time feedback using embedded, privacy-conscious sensors. Specifically, there is an unmet need for technically viable systems capable of supporting everyday behaviors—such as task transitions, routine recovery, or the expression of emotional states—without requiring explicit user input or wearable hardware. To illustrate, studies have highlighted the effectiveness of technology-based self-monitoring systems in supporting on-task behavior for students with disabilities [8], underscoring the potential for technology to foster self-regulation in vulnerable populations.

Importantly, this need becomes more pronounced in the context of vulnerable children experiencing care deficits. Recent research highlights that stressors in the family environment, such as those observed during the COVID-19 pandemic, can significantly disrupt children’s routines and emotional regulation, exacerbating developmental challenges [9]. Thus, designing systems that passively and ethically support routine formation, emotional expression, and behavioral transitions in the home becomes a pressing need. Furthermore, the successful development of technology-enhanced programs for child disruptive behavior disorders underscores the clinical relevance of such interventions [10]. As Bronfenbrenner’s ecological model of development emphasizes [11], children’s experiences are shaped by multi-layered, interacting environments from the immediate home to broader care structures. Ambient sensing systems must be designed in ecological alignment with children’s lived realities, not in isolation. The objectives of this paper are threefold:

1. To translate behavior-guided spatial strategies into implementable system logic.
2. To compare feasible sensing architectures in terms of behavioral resolution, privacy, and spatial adaptability.
3. To propose an integrated, low-cost sensing model suitable for compact child-centered environments.

Ultimately, this approach aims to establish a comprehensive design-to-technology framework that seamlessly bridges routine-supportive spatial designs with responsive, ethically sound sensing infrastructures.

## 2. Background and Related Work

### 2.1. Spatial and Ambient Cues for Behavioral Support

The role of spatial environments in guiding behavior, especially in child-centered contexts, has been increasingly emphasized across architecture, HCI, and cognitive psychology. Spatial affordances—such as lighting, layout, and rhythmic cues—can influence not only attention and routine adherence, but also emotional regulation (Kopec, 2012) [12]. In educational and therapeutic settings, the design of the physical environment is found to affect a child’s ability to self-regulate and engage meaningfully with their surroundings (Obrusnikova & Cavalier, 2011) [13].

In architectural design, Mostafa (2008) advocated for “sensory zoning” in autism-friendly spaces, proposing the modulation of sensory input to promote predictability and comfort [1]. More recently, Almaz and Mohamed (2024) highlighted how non-verbal spatial cues could reinforce emotional well-being in sensory-friendly environments [2]. These insights align with Gibson’s (1979) theory of affordances, which describes how individuals perceive opportunities for action within their environment based on both spatial characteristics and their own capabilities [14]. When applied to child-focused spaces, affordances become essential to understanding how design can implicitly scaffold behavioral responses.

Moreover, the importance of spatial-emotional interaction is reinforced by the field of emotion regulation. Gross (1998) emphasized that the environment can serve as a critical context for modulating emotional expression and recovery, suggesting that spatial cues can be deliberately leveraged to guide regulation strategies [15]. Accordingly, spaces designed with embedded, responsive features may play a role not only in behavioral support but also in enhancing a child’s emotional autonomy. Specifically, intelligent environments have shown promise in supporting children’s activities, such as homework, by integrating smart objects and robotic assistance [16].

However, while such research validated the importance of environment-behavior interaction, it often remained static—focused on design intention rather than responsive adaptation. In HCI, ambient displays have been proposed to encourage healthy routines without explicit user input. Consolvo et al. (2009) explored how context-aware systems like UbiFit could subtly encourage physical activity through ambient visual feedback [17]. Yet these systems targeted adults and wearable platforms with limited application in passive, spatially embedded systems for children. The broader field also emphasizes that effective technology-based solutions for behavior monitoring must draw lessons from past experiences, particularly regarding user acceptance and ethical considerations [7].

### 2.2. Behavioral Sensing in Domestic Environments

Behavior detection in home environments has traditionally employed simple, low-resolution sensors such as Passive Infrared (PIR), pressure sensors, or ambient light detectors (Wilson & Atkeson, 2005) [18]. While effective for detecting presence or motion, these devices suffer from limited resolution and struggle with capturing nuanced behaviors like posture, immobility, or emotional expression. However, this groundwork has been extended to include the detection of anomalies in daily routines, for example, identifying deviations in the activity patterns of older persons in smart home settings using unobtrusive sensors [19]. Recent advancements have shown that even these environmental sensors can contribute to robust human activity recognition, for instance, in smart buildings for occupancy detection [20] or when integrated with sensor fusion techniques [21]. Cost-effective and privacy-preserving systems utilizing pressure sensors have also been developed for activity recognition in smart home applications [22].

**Figure 1:**
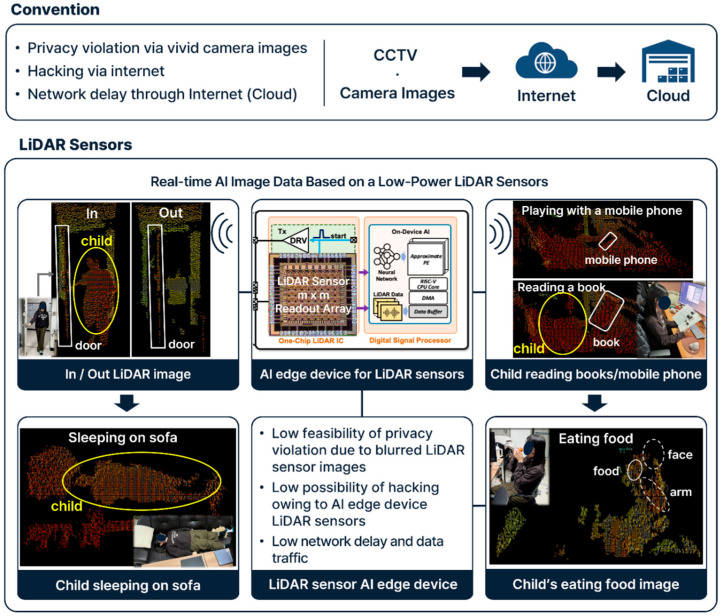
Advantages of LiDAR sensor AI edge device when compared to conventional data transfer through Internet.

LiDAR technology, particularly in Human-Activity-Recognition (HAR), has shown promise for higher-fidelity behavioral detection. Rinchi et al. (2023) reviewed LiDAR’s application in posture detection, movement trajectories, and room-level spatial mapping, emphasizing its non-contact advantages [23]. Alvarez et al. (2018) developed PeTra, a depth-sensing system using LiDAR and Kalman filtering to track limb motion, supporting privacy-conscious, real-time monitoring [24]. Furthermore, portable three-dimensional LiDAR systems have been developed for long-term and wide-area people behavior measurement, highlighting their utility in extensive spatial monitoring while maintaining privacy [25]. LiDAR point clouds are also being utilized to assess temporal behavior in complex environments like urban settings, showcasing their capability for dynamic behavioral analysis [26]. However, these applications are mostly found in robotics, automotive, or industrial contexts—not in compact, child-centered residential settings. Few studies explore how LiDAR can be used ethically and effectively for children in less supervised domestic environments. Figure 1 describes the advantages offered by a LiDAR sensor in domestic environments as an AI edge device, such as low privacy violation, low hacking possibility, and low network delay. It also shows some examples of image data taken from a LiDAR sensor, including a child coming in and out of a door, a child reading a book or a mobile phone, a child sleeping on a sofa, and a child eating food.

**Figure 2:**
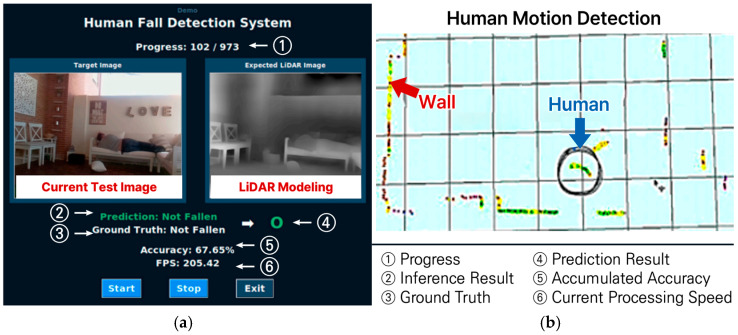
Applications of LiDAR sensors: (**a**) Fall detection [27]; and (**b**) Motion detection.

Figure 2a shows an application of a LiDAR sensor for human fall detection, where a LiDAR sensor image is compared with that of a camera. The average accuracy of a LiDAR sensor for fall detection could reach 68%, as described in Ref. [27]. Also, Figure 2b depicts a two-dimensional map of a human motion, where a real-time motion detection could be facilely acquired with a LiDAR sensor.

### 2.3. Privacy-Conscious and Ethical Sensing

The use of sensors in environments with vulnerable populations, such as children, raises significant ethical concerns. Traditional visual sensors (e.g., RGB cameras) are effective for fine-grained behavior recognition but pose severe privacy risks [28]. Alternatives, such as mmWave radar and thermal infrared (IR) sensing, have emerged as promising options for privacy-respecting monitoring. Zhao et al. (2021) demonstrated that mmWave radar could detect subtle human motions, such as breathing and posture changes, without capturing identifiable features [29]. Senstar Corporation (2023) introduced privacy-focused 3D LiDAR systems that processed motion data on the edge and avoided biometric storage, suggesting practical adaptations for home security [30]. The development of privacy-preserving Human Activity Recognition (HAR) systems, particularly for assisted living environments, highlights the feasibility of non-visual monitoring while maintaining dignity [31].

Recent advancements in mmWave radar specifically demonstrate its capability for fine-grained, environment-invariant behavior monitoring, such as identifying complex eating patterns without visual data [32]. It has also been successfully applied in pilot studies within real primary school environments to classify children’s activity levels [33]. Such systems leverage mmWave radar’s ability to operate effectively regardless of lighting conditions or occlusions, making them highly suitable for sensitive domestic settings.

However, the applications of such systems in behavioral routine support, especially for children, remain limited and underexplored.

### 2.4. Research Gap and Technical Challenges

Despite growing interest in emotion-aware and routine-supportive designs, few studies have connected spatial feedback systems to real-time, privacy-conscious sensor architectures in compact domestic environments. Existing works often separate design from sensing, therefore hindering the practical implementation of behavior-guided spatial systems due to limited technical translation between behavioral strategy and system logic. Moreover, the broader field of real-time human behavior monitoring still faces considerable challenges regarding data processing, accuracy in diverse environments, and ethical considerations [34]. This paper addresses this gap by:

1. Translating behavioral strategies into implementable sensing logics.
2. Evaluating sensor configurations in terms of privacy, fidelity, and spatial adaptability.
3. Proposing a technically feasible and ethically aligned sensing system for practical usage.

A critical challenge remains in the development of low-cost embedded sensor frameworks that operate without intrusive surveillance and support children’s autonomy and routine formation in sensitive domestic spaces. In particular, the system must not only detect behavior but enable children to build a sense of control over their own actions—what Bandura (1997) defines as self-efficacy [35]. Rather than treating users as passive targets of feedback, behavior-guided spatial systems should be designed to reinforce children’s perceived capability to initiate and regulate their own routines. This highlights the importance of aligning technical logic with psychological constructs to achieve developmentally supportive interaction models.

## 3. Design Framework and Technical Translation

This section presents a structured design framework that translates high-level behavioral intentions into implementable sensor-driven system logic. Developed to support children’s autonomy and emotional well-being, the framework consists of four core strategies that leverage ambient cues to gently guide behavior. Each strategy is transformed into a logic structure comprising input conditions, trigger thresholds, and feedback outputs. Then, this section concludes with a discussion of sensing modalities required for accurate and ethical system implementation.

### 3.1. Core Strategies for Behavior-Guided Design

Children experiencing environmental instability often face challenges in maintaining routines, expressing emotional states, and transitioning between tasks. The proposed framework responds to these needs through four strategies that rely on subtle environmental feedback rather than direct instruction. These strategies prioritize autonomy, reduce cognitive burden, and maintain dignity in everyday interactions. These strategies are deeply rooted in established developmental psychology and child-behavior literature, aiming to bolster the framework’s scientific and ethical foundation.

***Routine Recovery*** aims to help children resume missed (or delayed) activities by identifying the periods of inactivity during the expected routine times. This strategy employs time-based tracking in conjunction with motion sensing to detect deviations, for instance, when no movement is observed during the designated study periods. Upon detection, the system delivers subtle prompts such as ambient lighting changes or visual cues, thus encouraging re-engagement without creating pressure. This approach aligns with principles of behavioral consistency and the importance of predictable environments in reducing cognitive load and fostering a sense of security for children.

***Emotion-Responsive Adjustment*** acknowledges that children’s emotional states profoundly impact their engagement and well-being. This strategy focuses on validating expressed emotional states and fostering adaptive responses. It interprets various cues, including passive sensor data (e.g., prolonged stillness indicating distress) and child-initiated inputs (e.g., tactile buttons for emotional expression). The system then provides subtle, autonomy-supportive feedback like calming visuals or ambient color shifts. This strategy is informed by theories of emotion regulation (Gross, 1998 [15]), emphasizing how environmental cues can guide adaptive responses. It also leverages Bandura’s self-efficacy theory (Bandura, 1997 [35]) by encouraging children to actively engage in their emotional regulation and perceive control over their actions, rather than being passive recipients of feedback.

***Behavioral Transition Induction*** addresses the behavioral inertia caused by prolonged stillness or spatial fixation. This is identified through the indicators, such as a lack of posture change or extended presence in a single location. To gently encourage behavioral shifts, the system may activate light or sound cues that prompt children to move to another activity or spatial zone. This aligns with the concept of scaffolding, where environmental cues provide temporary support to help children transition between activities, thereby promoting independence and reducing resistance.

***External Linkage*** is activated when the system detects patterns of repeated disruption or distress. Utilizing simple pattern recognition algorithms, it identifies the thresholds that warrant external support, e.g., frequent help requests or sustained inactivity. Once triggered, alert messages are sent to caregivers or linked support systems to ensure timely intervention. This strategy is grounded in Bronfenbrenner’s ecological model of development (Bronfenbrenner, 1979 [11]), which highlights the critical role of broader ecological support systems (e.g., family, caregivers) in a child’s development and well-being, especially for vulnerable children experiencing care deficits. Altogether, these strategies prioritize autonomy over compliance. Rather than directing behavior, the system creates an environment that subtly responds to and supports the child’s actions, while preserving their dignity and agency.

### 3.2. Translating Design Strategies into System Logic

To enable practical implementations, each strategy must be translated into a system logic that can be interpreted by sensors and actuators, comprising three key components:

1. **Input Conditions**: What the system observes (e.g., lack of movement, emotional input).
2. **Trigger Thresholds**: When the system decides to act (e.g., inactivity longer than 15 min).
3. **Feedback Outputs**: How the system responds (e.g., ambient cue, notification, color shift).

**Figure 3:**
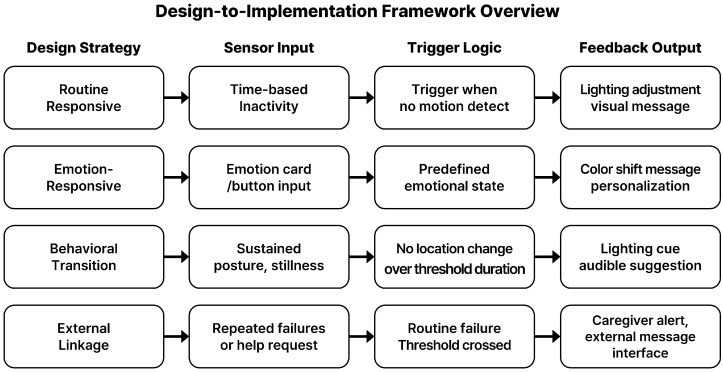
Translating behavior-guided strategies into sensor-based system logic.

**Figure 4:**
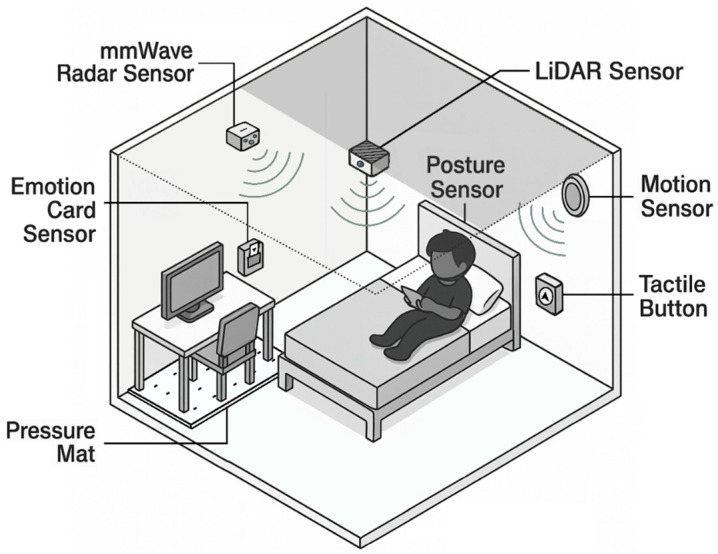
Sensor placement and spatial integration.

Figure 3 summarizes the core logic for each strategy. This structured logic is device-agnostic and can be adapted to different sensors (shown in Figure 4). However, the effectiveness of each logic pattern depends on the precision and context-awareness of sensor configurations.

### 3.3. Sensor-Centered Implementation

The effectiveness of behavior-guided spatial design lies not only in conceptual integrity, but also in its technical viability. To translate design strategies into functioning systems, we must bridge the gap between behavioral goals and sensor-based logic. This subsection outlines how each of the four core strategies introduced above maps onto specific sensing logics, emphasizing the role of real-time detection in enabling responsive environments.

#### 3.3.1. From Strategy to System Logic

Each design strategy, such as promoting recovery of missed routines or validating emotional states, requires a distinct mechanism for behavioral interpretation. This mechanism must capture the necessary level of detail while remaining respectful of the user’s autonomy, particularly in contexts involving children. We categorize the system logic into three functional types.

1. **Presence and Routine Detection** is used to assess whether expected behaviors (e.g., movement during routine times) are taking place. It supports strategies like ‘Routine Recovery’ and ‘External Linkage’.
2. **Posture and Stillness Interpretation** captures behavioral stasis or disengagement by recognizing prolonged stillness or inactive posture. It is relevant to ‘Behavioral Transition Induction’ and ‘Emotion-Responsive Adjustment’.
3. **Voluntary Feedback Capture** collects self-reported emotional cues through child-initiated inputs such as tactile buttons or emotion cards. This logic enhances the ‘Emotion-Responsive Adjustment’, thus offering ethical interaction without relying solely on passive sensing.

#### 3.3.2. Sensing Modalities and Their Roles

To support these logics, three broad categories of sensing modalities are deployed.

1. **Environmental Sensors** (e.g., PIR and pressure mats) detect presence or motion. These are discreet, low-cost, and minimally invasive, i.e., ideal for routine-based logic. Their ability to support Human Activity Recognition while being cost-effective and privacy-preserving, especially through pressure-based systems, has been demonstrated [22].
2. **Posture-Sensitive Sensors** (e.g., low-res LiDAR and mmWave radar) identify stillness, body orientation, and depth cues. These sensors enable higher-fidelity interpretation of behavioral disengagement. The fusion of various sensor types is critical to detect, track, and identify people in realistic scenarios, thereby enhancing overall system robustness [21].
3. **User-Triggered Inputs** (e.g., tactile interfaces) provide explicit emotional signals. They promote autonomy, reduce interpretive ambiguity, and avoid privacy concerns. This multi-modal sensing approach, particularly the fusion of passive data (e.g., from LiDAR or mmWave radar) with active, user-triggered inputs, is crucial for nuanced behavioral interpretation and respecting user autonomy. It allows the system to gather comprehensive information, covering both implicit behavioral cues and explicit self-reported states, especially vital for complex strategies like Emotion-Responsive Adjustment.

Each sensor type is mapped to a behavioral intention. For example, posture sensors and manual inputs may both contribute to interpreting emotional states, albeit through different channels, i.e., passive versus active.

#### 3.3.3. Evaluation Criteria for Sensor Suitability

Rather than selecting sensors by technical capability alone, behavior-guided systems must be evaluated through a four-dimensional ethical-operational lens. Table 1 describes the evaluation criteria in detail. These criteria serve as filters through which sensor choices must pass, depending on the strategy being implemented. No single sensor scores high in all categories, hence mandating hybrid configurations.

#### 3.3.4. Strategy-to-Sensor Mapping Summary

Table 2 summarizes how each strategy aligns with high-priority criteria and sensor types.

#### 3.3.5. Reframing Design as Interaction

**Figure 5:**
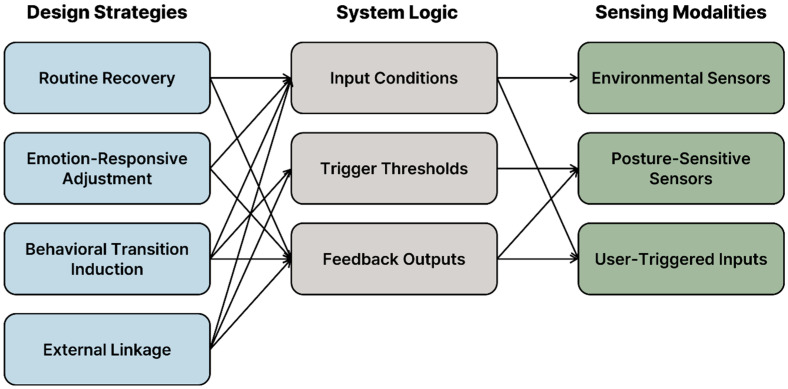
Design strategies, system logic, and sensing modalities in behavior-guided environments (arrows indicate logical relationships between conceptual design intentions and their sensor-based implementations).

This strategy-to-sensor mapping is more than a technical specification, because it represents a shift in how design is enacted. Spatial design becomes interactive infrastructure as shown in Figure 5, i.e., a conduit through which behavioral cues are received and interpreted in real-time, prompting calibrated environmental responses. Therefore, what begins as an abstract strategy becomes a real ambient interaction, where sensor systems are not passive monitors, but participatory agents that support care, autonomy, and engagement.

## 4. System Architecture and Workflow

Implementing behavior-guided spatial design in domestic environments requires not only a conceptual framework, but also a concrete system architecture that can interpret behavior, deliver feedback, and adapt over time. This section outlines the layered system structure, analyzes viable sensor configurations, details the feedback workflow, and explores spatial integration and ethical considerations necessary for practical implementation.

### 4.1. From Design Logic to System Architecture

**Figure 6:**
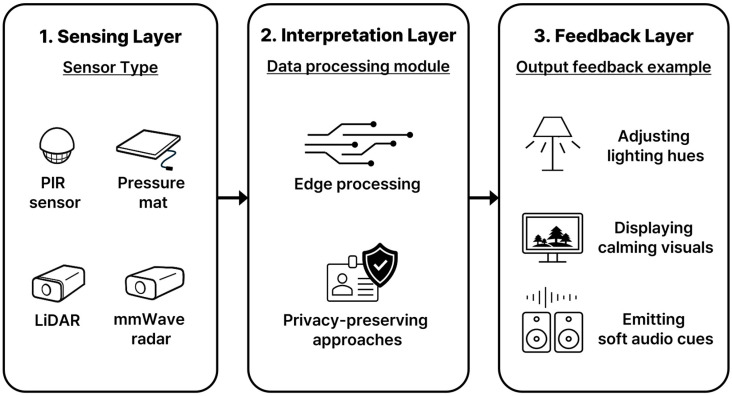
System architecture: Three-Layer Model.

To bring behavior-guided spatial design into practical use, abstract strategies must be grounded in a concrete system capable of sensing, interpretation, and responsive action. The proposed system architecture adopts a three-layered model that translates design logic into actionable technical functions (shown in Figure 6), while remaining compatible with domestic living environments and ethical constraints.

#### 4.1.1. Sensing Layer

**Figure 7:**
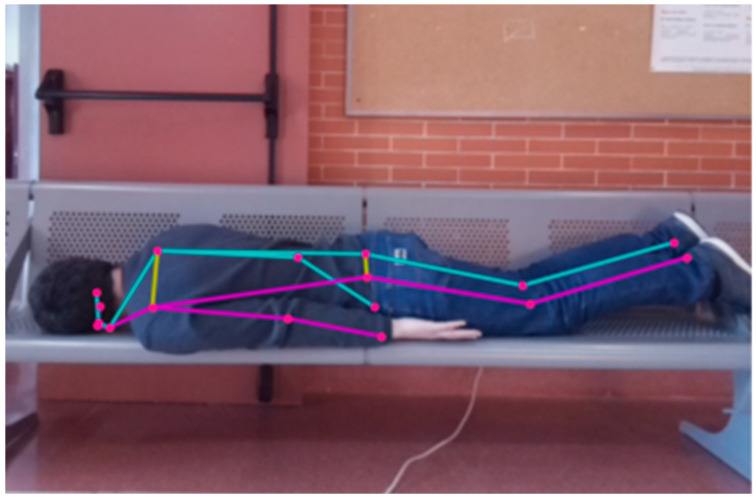
An example of skeletal joint data from a LiDAR sensor.

This layer includes embedded hardware components (such as passive infrared (PIR) sensors, pressure mats, LiDAR modules, or mmWave radar) that collect raw environmental and behavioral signals. These inputs include motion, presence, posture, and stillness, all of which serve as proxies for behavioral states. Raw data, such as LiDAR point clouds or mmWave radar return signals, are processed at this layer to extract minimal, non-identifiable features (e.g., bounding box coordinates and skeletal joint data as shown in Figure 7) before being passed to the interpretation layer. The design prioritizes unobtrusive placement and low maintenance, ensuring that the system can function passively without disrupting the daily rhythm of home life.

#### 4.1.2. Interpretation Layer

Raw sensor data (now in a privacy-preserved, feature-extracted format) are filtered and analyzed to infer behavior-related states, such as routine engagement, prolonged inactivity, or deviation from expected patterns. This layer incorporates rule-based logic and lightweight pattern recognition models to minimize computational overhead and enable real-time inference on the edge device. By avoiding cloud-based processing and complex biometric inference, the system respects users’ privacy while remaining adaptive. The interpretation logic maps directly to one of the four core design strategies introduced earlier.

#### 4.1.3. Feedback Layer

Based on the interpreted behavioral state, the system activates ambient feedback mechanisms, such as adjusting lighting hues, displaying calming visuals, or emitting soft audio cues. These outputs are designed to be non-intrusive and supportive, thus offering gentle prompts rather than corrective interventions. For strategies like Emotion-Responsive Adjustment, the system incorporates responses to child-initiated inputs (e.g., emotion cards or buttons), allowing for explicit communication and user-controlled feedback. This layer critically manages the fusion of passive sensor data (e.g., from LiDAR) and active, child-initiated inputs, ensuring the system’s responsiveness aligns with our ethical principles.

When interpreting a child’s emotional state, passive sensors provide ambient, non-identifiable cues like prolonged stillness or atypical posture that may suggest disengagement. Simultaneously, children can explicitly communicate their feelings through tactile buttons. Crucially, in cases where sensor data and child input suggest conflicting states (e.g., a passive sensor indicating distress while the child actively presses a “happy” button), our system strictly follows a “user-input priority” rule. This means the child’s explicit, voluntary input always takes precedence over passive sensor interpretations.

This design choice directly reinforces our core ethical commitment to respecting child autonomy and self-expression. Passive sensor data then serves to provide contextual background or to gently prompt the child for explicit input when direct communication is not initiated, empowering their agency rather than overriding it. The goal is to maintain user autonomy while nudging positive engagement or self-regulation.

These three layers form a closed-loop system that responds to real-time behavior and adapts based on short-term behavioral history. Moreover, the architecture emphasizes ambient intelligence over surveillance. Therefore, it avoids high-fidelity tracking or identity-based monitoring. Instead, it favors soft, spatial cues that encourage reengagement. This architectural model is designed with the practicalities of home life in mind, including compact spaces, shared usage, and ethical deployment for vulnerable populations (e.g., children). It lays the groundwork for integrating diverse sensor types and feedback mechanisms while maintaining a consistent behavioral logic across varied domestic settings. Section 4.2 below examines how this logic can be translated into practical technical configurations by comparing different sensing options, each with its own tradeoffs in resolution, privacy, and ease of integration.

### 4.2. Sensor Configuration Spectrum and Tradeoffs

No single sensor configuration can meet all the needs of behavior-guided spatial design. Instead, viable solutions emerge from balancing performance, intrusiveness, cost, and ethical compatibility. This section presents a spectrum of three representative sensors, each of which is optimized for different priorities and use contexts.

#### 4.2.1. Configuration Options

Table 3 describes the pros and cons of three different sensors, where options are not mutually exclusive. A hybrid configuration combining Options 1 and 2 may provide a practical balance between detection fidelity and privacy protection. As an example, an environmental sensor may detect presence, while a posture-sensitive sensor refines behavioral interpretation.

#### 4.2.2. Comparison by Key Evaluation Criteria

Table 4 shows three options for different criteria. This framework enables context-sensitive design. Below are some examples.

1. In a shared bedroom for siblings, Option 1 may be sufficient and least disruptive.
2. In a solo care room with emotional disengagement concerns, Option 2 or 3 may support subtle behavioral tracking better without visual monitoring.
3. Where ethical constraints prohibit any implicit monitoring, manual input tools (e.g., emotion cards) can complement sensing for respectful engagement.

#### 4.2.3. Design Implication

This spectrum-oriented approach recognizes that sensor integration is not a technical choice alone. It is a design decision shaped by architectural layout, caregiving context, and users’ comfort. Therefore, a successful implementation should allow for modularity and user-specific tuning, i.e., avoiding over-surveillance while still providing meaningful behavioral support.

### 4.3. Behavioral Feedback Workflow: A 7-Stage Adaptive Loop

**Figure 8:**
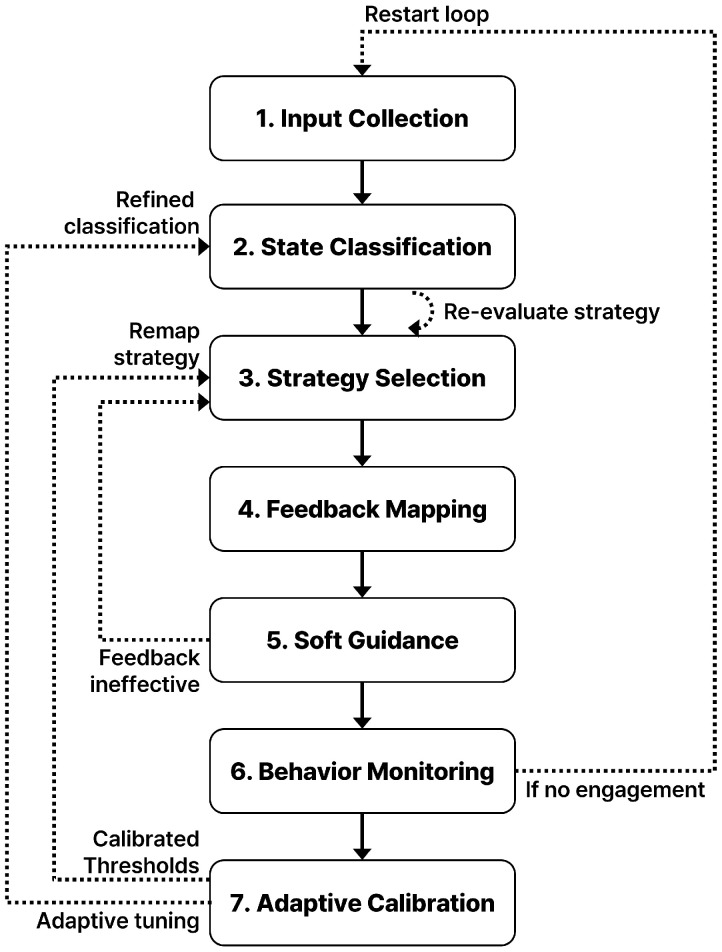
System Workflow Loop (7 Steps), illustrating the adaptive feedback mechanism.

To translate sensing configurations into meaningful support, the system must not only detect behavior, but also deliver timely and appropriate feedback. The following seven-stage loop (shown in Figure 8) describes how behavioral signals are processed and how the system responds within a child’s living environment. Designed for adaptability and ethical alignment, the loop avoids intrusive interventions and supports gradual, self-directed reengagement.

1. **Input Collection:** Behavioral signals such as movement, posture, stillness, or prolonged inactivity are continuously gathered through a configured sensor array, including passive infrared sensors, pressure mats, and posture-sensitive technologies.
2. **State Classification:** The system interprets raw input data to classify the current behavioral state. Predefined rules identify patterns such as activity, inactivity, routine disruption, or disengagement.
3. **Strategy Selection:** Based on the classified state, one of the four core strategies (Routine Recovery, Emotion-Responsive Adjustment, Behavioral Transition Induction, External Linkage) is selected to guide the system’s feedback logic.
4. **Feedback Mapping:** The selected strategy is translated into an ambient output plan, such as color-shifting lights, soft audio, or a glowing prompt, based on the pre-configured associations between strategy and sensory cues.
5. **Soft Guidance:** Feedback is delivered in a non-intrusive manner to nudge behavior gently, e.g., encouraging reengagement, facilitating activity transitions, or validating emotional expression. The system aims to support, not interrupt.
6. **Behavior Monitoring:** The system continues to monitor for signs of renewed activity or state resolution. If the intended engagement resumes, the system returns to a passive state, and the feedback is withdrawn.
7. **Adaptive Calibration:** Short-term behavioral data are stored locally to adjust timing thresholds and sensitivity parameters. This allows the system to refine its responsiveness over time while respecting privacy. Specifically, the system continuously analyzes localized, temporary behavioral logs (e.g., historical durations of activity/inactivity over a period) to dynamically tune trigger thresholds for each strategy. This prevents over-intervention by adjusting sensitivity based on observed patterns, fostering a more personalized and less intrusive supportive environment aligned with the child’s evolving rhythm. Adaptation can leverage simple statistical methods like moving averages or rule-based adjustments.

This feedback loop turns sensor data into compassionate interaction. Rather than commanding behavior, the system creates a responsive atmosphere that aligns with the child’s internal rhythm and autonomy. It avoids over-monitoring while maintaining structure, hence contributing to a home environment that quietly supports emotional and behavioral wellness.

### 4.4. Spatial Integration and Ethical Sensor Deployment

Effective implementation of sensing strategies depends on technical accuracy and thoughtful spatial integration. Each sensor type has distinct spatial characteristics, and their placement must align with both behavioral sensing goals and domestic privacy norms. Table 5 summarizes the recommended placements and the design considerations for each sensor type.

These placements aim to embed sensing seamlessly within the home, ensuring that the environment feels supportive rather than intrusive. Sensors should be visually discreet, physically unobtrusive, and integrated into objects or architectural elements wherever possible. Furthermore, ethical deployment requires avoiding any impression of surveillance. The goal is not to monitor users, but to quietly support their behavioral wellness through ambient cues. This principle helps to guide hardware placement, system calibration, and user communication. Section 4.5 below elaborates on the broader technical and ethical principles that support this human-centered sensing framework.

### 4.5. Technical Considerations and Ethical Design Principles

Creating a behavior-responsive environment for children requires more than functional sensing and feedback. It demands a foundation of ethical responsibility and technical restraint. The system must support behavioral well-being without intruding on privacy, compromising autonomy, or creating unnecessary dependency. Four guiding principles form the basis of this approach, as described in Table 6.

These principles ensure that the system acts as a quiet, respectful companion in the child’s daily rhythm and fosters reengagement and emotional regulation without requiring behavioral conformity or compromising personal space. With this ethical and technical foundation in place, the system becomes a viable, child-sensitive infrastructure for spatial care.

## 5. Comparative Evaluation of Sensor Architectures

### 5.1. Purpose and Evaluation Framework

To transition from design logic to real-world deployment, behavior-guided spatial systems must be supported by sensing architectures that should be technically sound, ethically responsible, and spatially feasible. This section builds directly on the layered system proposed in Section 4, offering a structured comparison of three viable sensing configurations, i.e., environmental sensors, low-resolution LiDAR, and mmWave radar. The goal is to understand how each option aligns with the four behavioral design strategies: Routine Recovery, Emotion-Responsive Feedback, Behavioral Transition Induction, and External Linkage. This approach recognizes that different domestic settings, user characteristics, and care goals may require different sensor logics. This comparative assessment goes beyond technical specifications by considering four interrelated dimensions.

1. **Detection Precision**: Can the system accurately detect and differentiate the key behavioral indicators, such as posture changes, immobility, or prolonged disengagement, at a sufficient resolution?
2. **Privacy Profile**: Does the system intrinsically preserve the dignity and autonomy of the user, particularly children in sensitive situations, by avoiding identifiable data capture and ensuring local processing?
3. **Spatial Integration**: Can the sensors be discreetly and easily deployed in everyday home environments without being obtrusive or stigmatizing?
4. **Implementation Feasibility**: Are the components affordable, readily available, and easily integrable with existing domestic technologies?

By framing the analysis through these dimensions, we aim to guide designers in selecting sensor types.

### 5.2. Strategic Fit and Selection Guidelines

This Section 5.2 consolidates the comparative findings across these dimensions and maps them onto the four design strategies to clarify where each configuration may be most effective. Also, this subsection synthesizes the practical and ethical characteristics of each sensor configuration and evaluates their suitability for implementing the behavior-guided strategies. The focus here is on how these sensing architectures align with practical priorities, such as ease of deployment, behavioral relevance, and user dignity.

#### 5.2.1. Overview of Sensor Options

Three sensor configurations are considered across the system. Although environmental sensors offer minimal intrusiveness and cost-effectiveness, they lack the ability to detect posture or prolonged inactivity. However, their utility in occupancy detection and basic activity recognition in smart buildings, sometimes augmented with fusion techniques, is well established [20,21]. Low-resolution LiDAR provides higher behavioral fidelity and adjustable privacy features. Yet, it mandates precise placement and moderate preprocessing. Its capabilities in human activity recognition, including posture and movement tracking for privacy-preserving monitoring, are widely reviewed [23,24,25,26]. Either mmWave radar or thermal IR excels in privacy and subtle motion detection, but presents algorithmic complexity and limited precedent in home settings. Recent studies highlight mmWave radar’s potential for environment-invariant micro-motion sensing and activity classification in real environments, even for children [32,33]. Each configuration involves tradeoffs along a spectrum of usability and ethical fit.

#### 5.2.2. Alignment with Behavioral Design Strategies

This analysis, summarized in Table 7, highlights how different sensor configurations align with our four design strategies. While each strategy has a primary mode of sensing (e.g., explicit input for emotion, or basic presence for routine), LiDAR and mmWave sensors serve as vital supplementary tools for nuanced behavioral interpretation. They provide passive detection of subtle behavioral cues like sustained stillness or changes in posture, which can indicate deeper emotional states or disengagement, thereby complementing direct inputs and offering more comprehensive and adaptive support, especially when explicit user input is not available or when confirming self-reported states.

Table 7 demonstrates that while all options can contribute to certain forms of feedback, LiDAR and mmWave radar are uniquely suited for strategies that require tracking nuanced behavioral shifts. Nonetheless, in low-risk or low-complexity contexts, environmental sensors may suffice, especially when paired with threshold-based decision logic.

### 5.3. Contextual Factors in Sensor Selection

Choosing a sensing configuration involves balancing technical precision with ethical and spatial realities.

1. **Low-resolution LiDAR** provides useful behavioral detail without compromising privacy and is especially suitable for shared domestic spaces like bedrooms and study areas where posture and motion inform engagement patterns. However, its performance can be affected by direct sunlight or highly reflective surfaces, requiring careful calibration.
2. **mmWave radar** excels in protecting privacy and detecting subtle movements even through obstacles. However, it requires higher algorithmic control for precise behavioral interpretation and is better reserved for future applications in sensitive care environments due to its current complexity and limited widespread precedent in consumer home settings
3. **Environmental sensors** provide a lightweight entry point for deployment, particularly where user familiarity and unobtrusiveness are priorities. Nonetheless, their functional range is narrow and better suited for routine pattern detection rather than fine-grained, real-time behavioral adaptation, primarily detecting presence or absence.

There is no one-size-fits-all configuration. The design strategy, user group, and spatial constraints must be considered in tandem to select the most appropriate sensing architecture.

### 5.4. Contextual Selection and Design Implications

**Figure 9:**
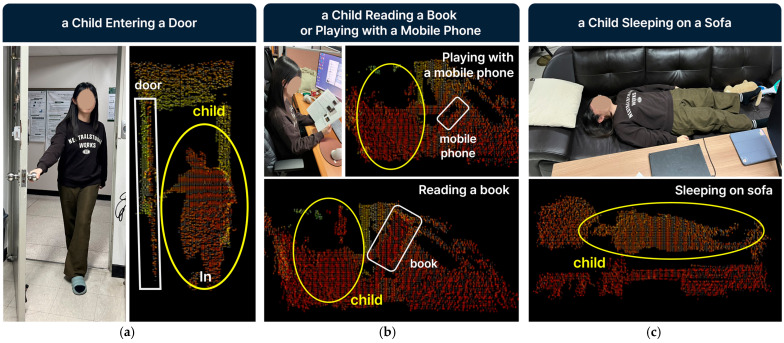
Comparison of a LiDAR sensor images with an RGC camera: (**a**) a child entering a door, (**b**) a child reading a book or playing with a mobile phone; and (**c**) a child sleeping on a sofa.

To practically evaluate the capabilities supporting our sensor selection, we conducted preliminary observations using a Hypersen HPS-3D160 LiDAR sensor, which utilizes Time-of-Flight (ToF) ranging technology. Its key technical specifications are presented in Table 8. Our experimental setup, designed to emulate realistic residential conditions, included furnishing an indoor environment with typical household items. The sensor data processing was performed on a laptop equipped with an 11th Generation Intel Core™ i7-1165G7 CPU (2.8 GHz, 4 cores, 8 threads). The operating system is Ubuntu 24.04 running on WSL2, and the development environment comprises Eclipse IDE 4.27.0, PCL 1.14, and C++. Figure 9 demonstrates the images of a child taken from an RGB camera and compares them with the LiDAR sensor images, explicitly illustrating the privacy-preserving nature of LiDAR data.

Selecting the appropriate sensing configuration for behavior-guided spatial systems demands careful balancing of behavioral relevance, ethical considerations, and spatial compatibility, particularly when applied to child-centered environments. Among the three configurations, low-resolution LiDAR emerges as the most balanced option for early-stage implementation because it offers sufficient details to support the full range of design strategies while maintaining a moderate level of privacy and technical feasibility. Our preliminary observations, conducted using the Hypersen HPS-3D160 LiDAR sensor in a simulated domestic environment, confirm that this LiDAR sensor effectively captures key behavioral indicators such as changes in posture, sustained stillness, and presence/absence from defined zones with a high degree of fidelity. This is achieved while inherently preserving privacy by rendering individuals as non-identifiable point clouds (as exemplified in Figure 9). This practical capability, combined with its compact size and compatibility with edge processing, strongly supports its prioritization.

With its proper calibration and privacy-preserving measures (such as skeletal reduction and local processing), LiDAR can be deployed effectively in shared domestic spaces like bedrooms or study areas, where posture and immobility are key behavioral cues. Meanwhile, mmWave radar is a promising solution in terms of privacy protection and subtle motion detection. However, it presents greater algorithmic complexity and lacks widespread precedent in residential settings. Its deployment may be more suitable in future applications or in highly sensitive care contexts, such as trauma-informed shelters, where facial anonymity and non-visual sensing are priorities. Environmental sensors remain the most practical in terms of cost and unobtrusiveness. Though limited in precision, they can still play a valuable role in routine-based feedback or layered configurations that rely on threshold logic. Their simplicity and ease of deployment make them appropriate for entry-level installations or supplementary usage in combination with other sensors.

Consequently, sensor architecture is a design decision. Each configuration reflects a particular stance on what matters most: behavioral granularity, spatial subtlety, ethical transparency, or implementation practicality. Designers must navigate these tradeoffs based on the specific goals, constraints, and sensitivities of their target environment.

## 6. Toward Empirical Validation: Prototype and Research Plan

To explore how the proposed system might function in everyday settings, this section outlines a pilot implementation plan. The goal is to assemble accessible, privacy-respecting components into a working prototype that reflects the core design strategies.

### 6.1. Prototype Configuration and Use Scenario

The prototype will use a low-resolution LiDAR sensor combined with a local edge-processing unit. This setup is intended to detect the key behavioral patterns, including posture, immobility, and spatial absence, without capturing or storing visual or biometric data. The system will operate entirely on-device, with no cloud connection or data upload.

A typical scenario involves a child’s bedroom or study area. When the system detects the behavioral cues suggesting disengagement, such as sustained stillness or absence from a defined zone, it will respond with subtle visual feedback, like a change in ambient lighting or the appearance of a symbolic icon. For strategies like Emotion-Responsive Adjustment, the prototype will additionally incorporate child-initiated inputs, such as tactile buttons or emotion cards, thus allowing children to explicitly communicate their emotional state. This gentle interaction aims to prompt re-engagement, facilitate activity transitions, or trigger caregiver awareness, all without disrupting the child’s autonomy or relying on intrusive sound/verbal messages unless initiated by the child. Table 9 summarizes the various features of the LiDAR sensor prototype.

### 6.2. Planned Pilot and Evaluation Parameters

A short-term pilot study is planned to assess how the system performs under real domestic conditions. Although this work is conceptual at this stage, it is designed to reflect realistic deployment constraints and ethical priorities. This pilot aims to provide a proof-of-concept for our system, akin to studies validating anomaly detection in smart homes for vulnerable populations [19] or technology-enhanced programs for child behavior disorders [10].

1. **Participants**: Fifteen to twenty households with children aged six to ten.
2. **Location**: Bedrooms, study nooks, or shared domestic play areas.
3. **Duration**: One month per household (four weeks).
4. **System Goals**:
  A. Detect presence, absence, and posture changes in daily routines.
  B. Trigger ambient, child-appropriate feedback without capturing identifiable data.
  C. Operate reliably with minimal maintenance.

Evaluation will focus on three dimensions. First, detection accuracy will be quantitatively assessed by using metrics (such as precision, recall, and F1-score) and comparing system logs with caregiver reports. Second, operational stability, whether the system functions consistently across varied room conditions, is evaluated. Third, social fit, including how the feedback was received by children and whether it influenced behavior in nonintrusive ways, will be estimated through qualitative measures such as post-pilot interviews with caregivers and children (where developmentally appropriate), and analysis of observed interactions. A clearly defined baseline is crucial for robust evaluation. Our pilot study will initially employ a “no system” baseline, comparing observed child behaviors and caregiver reports during periods without the system to those with the system active within the same households. This approach aims to establish the system’s fundamental effectiveness and user acceptance in a real-world domestic context. While our current pilot focuses on this baseline, we acknowledge the value of comparing our LiDAR-based approach against simpler systems (e.g., basic PIR sensors and timers) to demonstrate its unique advantages. Such comparative studies will be a crucial component of future, larger-scale empirical research, building on insights from this initial pilot.

Findings from this pilot will inform iterative refinements to the system for enhanced applicability within diverse domestic environments. This includes improving its adaptability to various home layouts, user demographics, and specific routine challenges. Crucially, while this integrated pilot focuses on overall system feasibility and acceptance, future, more rigorous studies will aim to investigate the isolated effects and comparative contributions of individual strategies (e.g., Routine Recovery vs. Behavioral Transition Induction) for a deeper understanding of component efficacy. Furthermore, these insights will lay the groundwork for potential future adaptations and explorations into specialized contexts, such as deployment in child welfare facilities, trauma-informed shelters, or modular educational units. While the current version uses a LiDAR sensor for its balance of precision and privacy, later iterations may incorporate mmWave radar or alternate sensors depending on users’ feedback and implementation needs. Table 10 shows the details of different deployment constraints.

## 7. Conclusions

This paper presents a robust conceptual framework and a working system architecture for a non-intrusive spatial system designed to support child behavior in domestic environments. We first defined four core behavior-guided design strategies—routine recovery, emotion-responsive adjustment, behavioral transition induction, and external linkage—and meticulously translated each into detailed system logic outlining input conditions, trigger thresholds, and feedback outputs. Our comparative conceptual analysis identified low-resolution LiDAR as the most balanced sensing configuration, offering sufficient behavioral insight while enabling edge-based local processing, robust privacy protection, and compatibility with compact residential settings. Preliminary technical observations from lab-based sensor tests using a Hypersen HPS-3D160 LiDAR (specifications detailed in Table 1) support this selection, confirming its practical capability to capture key behavioral indicators while preserving privacy. The proposed three-layered system architecture emphasizes edge processing and minimal-intrusion feedback, aligning with principles of child autonomy and ethical design.

The primary contribution of this work lies in its holistic translation of ambient behavior-guiding concepts into actionable system logic and a feasible implementation roadmap. Rather than inventing new technologies, this work prioritizes adapting and integrating accessible tools to bridge the gap between design intent and real-world deployment. Our detailed pilot implementation plan (Section 6), tailored for small-scale home environments, outlines a concrete path for future empirical validation of system effectiveness and user acceptance. This structured design approach and pilot framework lay a crucial foundation for broader applications in diverse residential and care contexts, facilitating longitudinal observation of behavioral patterns and iterative refinement.

While this paper establishes a strong foundation, the comprehensive empirical validation of the system’s real-world effectiveness and performance is the next critical phase of our research. Our claims, while theoretically well grounded and supported by initial technical observations, require rigorous empirical substantiation through broader deployments. Future work will involve fine-tuning specific algorithms for nuanced behavioral interpretation (e.g., precise posture recognition) and adaptive calibration based on empirical data. Furthermore, extending the system to multi-child households and larger, open-plan environments presents significant scalability challenges, demanding advanced individual distinction, conflict management, and comprehensive sensor fusion techniques. A crucial ethical consideration for future research also involves continuously monitoring the potential for unintended psychological consequences, such as over-dependence on environmental cues hindering internal self-regulation, which will be addressed through mechanisms like adaptive fading of cues and continuous assessment of child autonomy.

Ultimately, this work contributes to the broader discourse on how technology can ethically and developmentally support children’s autonomy and well-being, moving beyond surveillance to enable subtle, ambient, and socially responsible spatial interactions attuned to children’s everyday lives.

## Figures and Tables

**Table 1:** Four evaluation criteria.

Evaluation Criteria	Description
Privacy Sensitivity	Level of intrusiveness and potential for re-identification; assessed by: (1) Identifiability of raw data (e.g., facial features), (2) Level of data abstraction (e.g., point cloud vs. bounding box), (3) Processing location (edge vs. cloud), and (4) Data retention policy.
Detection Fidelity	Accuracy (precision, recall, F1-score) in recognizing key behavioral indicators (e.g., posture shifts, sustained stillness) and differentiating subtle human activities.
Spatial Integration	Ease of physical deployment (e.g., installation complexity, calibration requirements), aesthetic compatibility with domestic settings, and minimal physical disruption.
Feedback Synchronization	Timeliness of system response (latency from input to output) and contextual relevance of feedback based on real-time sensor data, ensuring effective behavioral nudges.

**Table 2:** Four different strategies with high-priority criteria.

Strategy	High-Priority Criteria	Preferred Sensor Types
Routine Recovery	Feedback Synchronization, Spatial Integration	PIR, pressure mat
Emotion-Responsive Adjustment	Privacy, Detection Fidelity	Emotion card, mmWave radar
Behavioral Transition Induction	Detection Fidelity, Feedback Synchronization	mmWave radar, low-res LiDAR
External Linkage	Privacy, Spatial Integration	PIR, tactile button

**Table 3:** Pros and cons of three different sensors.

Option	Sensor Types	Key Advantages	Key Limitations
1	Environmental sensors (PIR, pressure mat, ambient light)	Low cost, easy installation, privacy-preserving	Cannot detect posture or nuanced stillness
2	Low-resolution LiDAR	Captures spatial engagement, posture, and inactivity accurately	Requires careful placement and calibration
3	mmWave radar, thermal IR	Works in low light, enables non-visual tracking, protects privacy	High algorithmic complexity, limited deployment history

**Table 4:** Three options for different criteria.

Criteria	Option 1	Option 2	Option 3
**Privacy**	High	Moderate	High
**Detection** **Fidelity**	Low	Moderate to high	High
**Installability**	Very high	Moderate (requires clear space)	Moderate to low (sensitive to layout)
**Feedback** **Timing**	High (fast and simple signals)	High	High (if algorithms are tuned well)

**Table 5:** Design considerations of different sensors.

Sensor Type	Recommended Location	Design Considerations
PIR	Entrances, desks	Detects transitions between spaces; should avoid persistent monitoring zones
Pressure Mat	Beds, chairs	Embedded in seating or bedding surfaces; must remain unobtrusive and passive
Light Sensor	Ceilings, upper corners	Used to monitor ambient brightness; should not interfere with visual comfort
LiDAR	Ceiling-mounted, angled downward	Requires careful cone-of-view design to avoid facial recognition or direct gaze
mmWave Radar	Upper wall, high corners	Non-visible; maintains privacy even in dark; requires directional calibration

**Table 6:** Four guiding principles for the proposed system.

Principle	Description
Edge Processing	All data is processed locally within the system device. No cloud connectivity is used, ensuring that behavioral data remains within the child’s space.
No Biometric Capture	The system avoids cameras, microphones, or any biometric sensors that could identify or surveil the child. This maintains dignity and eliminates profiling risks.
Temporary Data Use	Behavioral logs are stored only temporarily to allow adaptive calibration. No long-term personal data is retained.
Informed Consent	The system operates transparently. Where developmentally appropriate, children are informed of how the system works and are given the option to engage.

**Table 7:** Analysis of four different design strategies and their sensor fit.

Design Strategy	Sensor Fit	Justification
Routine Recovery	All options viable	Basic presence/inactivity detection is sufficient
Emotion-Responsive Adjustment	LiDAR or mmWave	Captures behavioral indicators for emotional states; complements direct input
Behavioral Transition Induction	LiDAR or mmWave	Requires detection of immobility or spatial stagnation
External Linkage	All (if logic applied)	Triggered by repeated absence or failure patterns; logic-based rather than resolution-based

**Table 8:** Key Technical Specifications of HPS-3D160 LiDAR Sensor.

**Parameter**	Values	Unit
Size	78 (L)×40 (W)×30 (H)	mm
Weight	110	g
Power supply	9~12	V
Maximum power consumption	6	W
Operating temperature	−10~55	°C
Infrared VCSEL emitter	850	Nm
Emitting angle	$76\left(Horizontal\right)\times 32\left(Vertical\right)\;$	°
Maximum measurable distance	12	m
Minimum measurable distance	0.25	m
Maximum output frame rate	35	fps

**Table 9:** Summary of prototype features.

Category	Details
Sensor Setup	Low-resolution LiDAR with a local edge processor (e.g., Raspberry Pi, Jetson Nano)
Core Functions	Detects posture, immobility, presence; operates in real-time; no image or biometric storage
Feedback Modality	Visual cues only; color-shifting lights, ambient icons (no sound unless user-triggered)
Target Spaces	Shared domestic settings (bedrooms, study areas, play zones)
Design Principles	Privacy-preserving (non-visual sensing), Seamless spatial integration, User-controllable feedback

**Table 10:** Deployment constraints for evaluation and future research.

Category	Details
Participant Households	6 to 10 families with children aged 6–10
Installation Areas	Daily-use child spaces (e.g., bedroom, desk area)
Trial Duration	Two weeks per household
Evaluation Criteria	Detection accuracy (vs. caregiver logs), System reliability (uptime, fault tolerance), Social fit (child response, behavioral influence)
Future Expansion	Applicable to group housing, welfare shelters, learning spaces Potential for mmWave integration in sensitive environments

## Data Availability

Data is contained within the article.
